# P-163. Efficacy of Wolbachia-mediated sterility to suppress dengue

**DOI:** 10.1093/ofid/ofae631.368

**Published:** 2025-01-29

**Authors:** Jue Tao Lim

**Affiliations:** LKCMedicine, NTU, Singapore, Singapore

## Abstract

**Background:**

Vector control remains the primary means to mitigate the spread of dengue. Matings between male Aedes aegypti mosquitoes infected with the wAlbB strain of Wolbachia and wildtype females yield non-viable eggs (Wolbachia-Aedes). We evaluated the efficacy of Wolbachia-Aedes to suppress dengue incidence. We present data comprising a field trial of 607,872 residents over 5 years.

**Methods:**

We utilized the synthetic control method and trial emulation approach to evaluate large-scale field trials in Singapore involving the deployment of Wolbachia-Aedes for dengue control via vector population suppression, from epidemiological week (EW) 27, 2018, to EW 26, 2022. We selected two large towns (Yishun and Tampines) to adopt an expanding release strategy and two smaller towns (Bukit Batok and Choa Chu Kang) to adopt a targeted-release approach. All intervention and control locations practised the same baseline dengue control protocol. The main outcome was weekly dengue incidence rate caused by any dengue virus serotype (in the case of the synthetic control method) and risk of being test positive for dengue (when using the trial emulation approach). We used incidence data collected by the Singapore Ministry of Health as well as a nationally representative dengue test positive/negative database to assess the efficacy of the interventions, To compare interventions, we used the synthetic control method or constrained randomization protocols to generate appropriate counterfactuals for the intervention towns selected from a combination of 30 control towns from EW 1, 2014 to EW 26, 2022.

**Results:**

Our study comprised an at-risk population of 607 872 individuals living in intervention sites and 3 894 544 individuals living in control sites. Interventions demonstrated up to 77⋅28% (121/156, 95% CI 75⋅81–78⋅58) intervention efficacy despite incomplete coverage across all towns. Intervention efficacies increased as release coverage increased across all intervention sites. Secondary analysis showed that these intervention effects were replicated across all age groups and both sexes for intervention sites.

**Conclusion:**

Wolbachia-mediated incompatible insect technique can strengthen dengue control in tropical cities, where dengue burden is the greatest.

**Disclosures:**

**All Authors**: No reported disclosures

